# Supplementary material for: Causal interplay between lactose intolerance and gut microbiota: a combined bidirectional Mendelian randomization and in vivo validation study
Source: Front Nutr. 2026 Jun 1;13:1803337. doi: 10.3389/fnut.2026.1803337 (PMC13265576; doi:10.3389/fnut.2026.1803337)
Supplement: Supplementary file 1 [file Data_Sheet_1.zip › supplementary materials/Forward/leave one out cross validation/ebi-a-GCST90027654.finngen_R12_E4_LACTONAS.pdf]

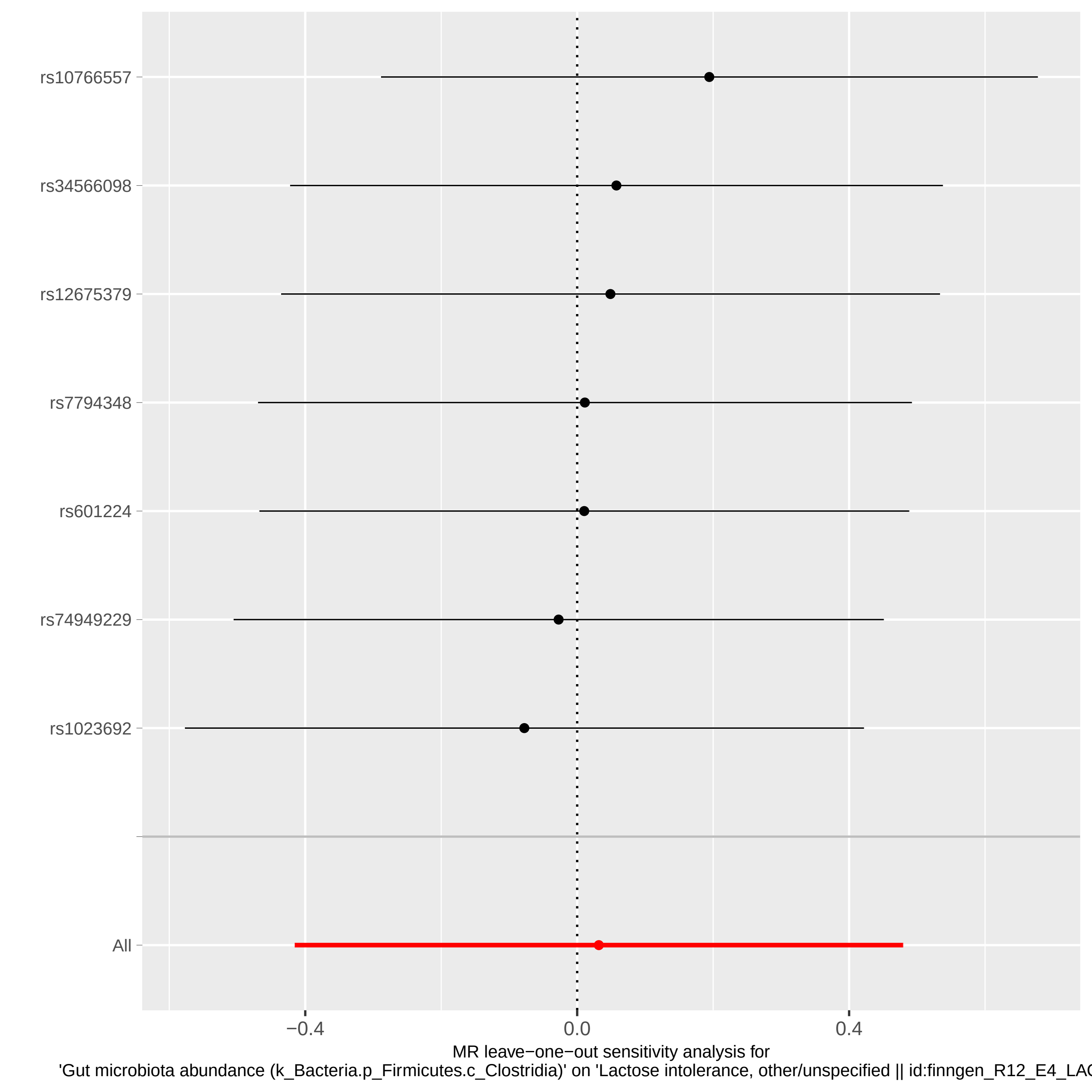

rs10766557
rs34566098
rs12675379
rs7794348
rs601224
rs74949229
rs1023692
All
−0.4
0.0
0.4
MR leave−one−out sensitivity analysis for
'Gut microbiota abundance (k_Bacteria.p_Firmicutes.c_Clostridia)' on 'Lactose intolerance, other/unspecified || id:finngen_R12_E4_LA
